# Commentary: Neurobiology and Therapeutic Potential of Cyclooxygenase-2 (COX-2) Inhibitors for Inflammation in Neuropsychiatric Disorders

**DOI:** 10.3389/fpsyt.2020.00264

**Published:** 2020-04-22

**Authors:** Clara Westwell-Roper, S. Evelyn Stewart

**Affiliations:** ^1^Department of Psychiatry, Faculty of Medicine, University of British Columbia, Vancouver, BC, Canada; ^2^British Columbia Children's Hospital Research Institute, Vancouver, BC, Canada; ^3^BC Mental Health and Substance Use Services Research Institute, Vancouver, BC, Canada

**Keywords:** innate immunity, inflammation, obsessive compulsive disorder, child and adolescent psychiatric disorders, COX-2 inhibitor, immunopsychiatry, prostanoid, non-steroidal anti-inflammatory drug

## Introduction

Enzymes of the cyclooxygenase (COX) family catalyze the metabolism of arachidonic acids to prostanoids. In the central nervous system (CNS), COX-1 is constitutively expressed by neurons, astrocytes, and microglia; COX-2 is expressed by glutamatergic neurons in the cerebral cortex, hippocampus, and amygdala and is inducible in other cell types (1, 2). COX-2 and its products play important physiological role in synaptic plasticity and long-term potentiation but may also contribute to neuropathology by enhancing glutamate excitotoxicity, promoting neuronal cell death, and oxidizing endogenous cannabinoids (3, 4). Some studies suggest upregulation of COX-2 expression in inflammatory and neurodegenerative diseases as well as schizophrenia and bipolar disorder (1). Non-steroidal anti-inflammatory drugs (NSAIDs) inhibit COX enzymes either selectively or non-selectively. In rat models, selective COX-2 inhibitors such as celecoxib inhibit microglial activation (5) and glutamate release (6) and enhance serotonergic and noradrenergic output in the prefrontal cortex (7, 8). Meta-analyses suggest possible benefit of adjunctive COX-2 inhibitors in the treatment of major depressive disorder (MDD) (9) and first-episode psychosis (10, 11); the general role of immunomodulation in these disorders has been recently reviewed (12).

**Figure 1:**
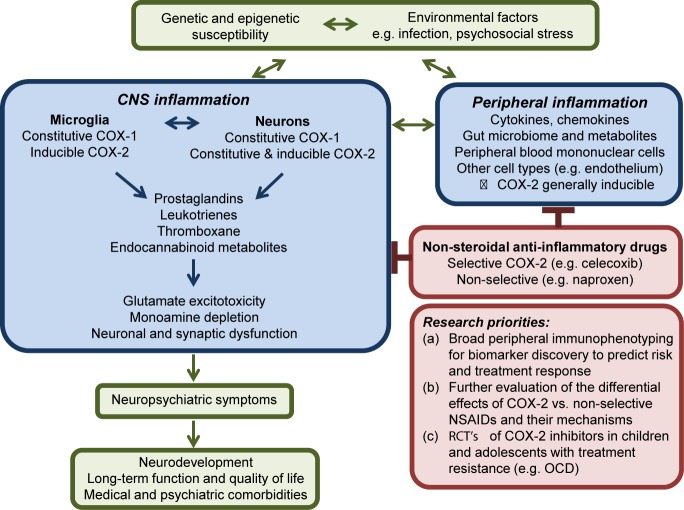
Simplified schematic overview of COX activity in the central nervous system in psychiatric disorders. The inflammatory response may represent one mechanism by which environmental factors including psychosocial stress contribute to the development or perpetuation of neuropsychiatric symptoms in individuals with underlying genetic susceptibility. While typically inducible in other cell types, COX-2 is expressed constitutively at post-synaptic membranes by groups of neurons in the prefrontal cortex, hippocampus, and amygdala. Release of prostanoids that act on pre-synaptic receptors may contribute to dysfunctional synaptic remodeling, altered calcium homeostasis, glutamate excitotoxicity, and neuronal cell death. Research priorities are further described in the text.

Sethi and colleagues provide an important and timely review of pre-clinical and clinical studies investigating the use of COX-2 inhibitors across multiple psychiatric disorders including major depressive disorder, schizophrenia, bipolar affective disorder, autism spectrum disorder (ASD), and obsessive compulsive disorder (OCD). Other than a clinical trial protocol for celecoxib as an adjunct to vortioxetine in depression published in 2018 (13), their review of randomized controlled trials (RCT's) through November 2017 remains up to date 2 years later. In this commentary, we highlight three factors arising from their results that are essential to advancing this research agenda. First, there is a critical need to move beyond schema that use individual markers to characterize peripheral “pro-” and “anti-” inflammatory states, as well as M1/M2 microglial polarization. This model has the potential to vastly oversimplify the role of innate immunity in brain homeostasis and to limit biomarker discovery efforts. Second, COX inhibitors have direct and indirect effects on the functions of both neurons and glia. COX selectivity as well as COX-independent mechanisms of action vary among NSAIDs. More work is needed to determine which drugs of this class are the best candidates for adjunctive therapy targeting specific neuropsychiatric symptoms. Finally, childhood-onset neuropsychiatric symptoms represent important targets for early intervention with low-risk therapies, but there is little evidence to inform their use. **Figure 1** outlines the potential role of COX signalling in psychiatric disorders and related research priorities.

## Measuring Inflammation in the Brain and Periphery: Beyond Polarization

The identification of immune-related bio-signatures will ideally assist in predicting risk of disease, prognosis, and response to therapy (14). Sethi et al. describe elevated pro-inflammatory markers as “consistently associated with neuropsychiatric symptoms.” While promising studies have begun to identify subpopulations of patients with MDD likely to respond to anti-inflammatory therapy (15), results of immune phenotyping in other disorders have been variable. A recent systematic review found that meta-analyses for MDD, ASD, bipolar disorder, and schizophrenia have consistently reported changes in only 16, 7, 8, and 7 individual inflammation-related factors in peripheral blood, respectively (16). The single meta-analysis of immune phenotyping studies in OCD was filtered out because of insufficient statistical power (16). Longitudinal data were lacking and state *versus* trait markers difficult to distinguish, markers were restricted to a few per study based on a biased candidate gene/cytokine approach, and the contribution of confounding factors—including childhood adversity, diet, and smoking—was potentially significant (16). Ultimately, longitudinal clinical characterization combined with a broad approach to immune phenotyping—as employed in a recent analysis of microarray data in MDD (17)—is likely a higher-yield approach both for biomarker discovery and for improving our understanding of how peripheral inflammation reflects or perpetuates psychiatric symptoms.

Sethi et al. also provide a common yet limiting perspective on microglial activation states. The disadvantages of the M1/M2 conceptualization of macrophage (18) and microglial (19) polarity *in vivo* have been previously discussed. As an *in vitro* construct that relies on stimulating cultured cells with a defined set of factors (20), its application to *in vivo* conditions is generally limited (21). Moreover, so-called M1 and M2 gene signatures often coexist in complex mixed phenotypes; the dichotomous paradigm is not supported by transcriptional profiling of human macrophages or monocytes activated by diverse ligands (22). Emerging evidence suggests that microglial subtype categorization should consider both their environment-dependent plasticity and subtypes with inherent functional specificity (23). Technologies that can assist in these efforts include two-photon imaging, whole-genome transcriptomic and epigenomic analyses at the cellular level, mass cytometry, and high-content experimental models (19).

Evidence for activation of microglia in patients with psychiatric disorders does not appear to be specific for any particular diagnostic category. *Post-mortem* characterization of microglia together with evaluation of translocator protein positron emission tomography imaging in patients with MDD suggest that the severity of illness—marked by limited response to traditional medications and increased suicidality—rather than the presence of the disorder itself is associated with altered microglial phenotypes (24, 25). This has led to the hypothesis that inflammation in the CNS primarily reflects psychological stress (26). COX inhibition may therefore be most effective for individuals with severe pathology of diverse etiologies, and when employed at the earliest clinical stage possible may alter the path toward chronic aberrant innate immune activity.

## Diverse Non-Steroidal Anti-Inflammatory Drug Mechanisms of Action: The Specific Drug Matters

While all NSAIDS have anti-inflammatory, antipyretic, and analgesic properties attributable to prostaglandin inhibition, they vary with respect to COX selectivity (27) and likely with respect to COX-independent mechanisms (28–30). In the CNS, modulation of glutamate, serotonin, norepinephrine, and endocannabinoid signaling has been demonstrated for COX-2 inhibitors, while the role of non-selective NSAIDs in neurotransmitter function is less clear (3, 4, 6–8). NSAID use has also been associated with distinct gut microbial populations (31), an additional mechanism by which this class of drugs could affect neural development, cognition, and behaviour (32).

Few RCT's have evaluated non-selective NSAIDs in primary psychiatric disorders, although the literature includes a negative RCT of naproxen in geriatric depression (33) and a study of adjuvant aspirin in schizophrenia suggesting some benefit (34). Clinical practice guidelines for the treatment of children with pediatric acute-onset neuropsychiatric syndrome (PANS) and pediatric autoimmune neuropsychiatric disorder associated with streptococcal infection (PANDAS) recommend the use of naproxen before celecoxib because of its “greater potency” (35), despite clinical studies showing benefit of adjunctive celecoxib in OCD (36, 37) and pre-clinical data demonstrating celecoxib-mediated enhancement of the serotonergic effects of fluoxetine in a rat model of anxiety (38). Moreover, observational studies have focused on NSAIDs as a class in children with PANS/PANDAS (39, 40). Given the significance of different COX isoforms and their unknown relative “potencies” in the CNS, careful attention must be given to selection and evaluation of specific NSAIDs.

## Need for Pediatric Studies and Early Intervention

A recent Danish population-based study suggested that environmental factors related to infection and inflammation are associated with the development of multiple mental disorders in children (41), adding to growing support for the link between immune activity and psychiatric symptoms early in life. However, there is currently little evidence to inform the use of adjunctive anti-inflammatory agents in children and adolescents with psychiatric disorders. Studies of peripheral inflammatory markers in this population have been equivocal, largely limited by similar methodological factors as adult studies (42, 43).

Early-life stress is more clearly associated with overt inflammation prior to the development of neuropsychiatric symptoms. For example, childhood trauma is associated with significantly elevated peripheral levels of C-reactive protein, interleukin (IL)-6, tumor necrosis factor-a, and soluble urokinase plasminogen activator receptor (44, 45). Elevated IL-6 in childhood is in turn associated with increased risk of future depressive and psychotic symptoms in adolescence (46, 47). Stress-related epigenetic dysregulation in immune networks represents one mechanism by which childhood experiences may become biologically embedded (48), and a potential target for early intervention. Epigenetic modifications facilitate the phenotypic plasticity of macrophages, are critical to their role in maintenance of tissue homeostasis, and contribute to a form of innate immune “memory” that persists across the lifespan (49, 50).

Randomized controlled trials of COX-2 inhibitors as adjunctive therapies in children with treatment-resistant psychiatric disorders with a potential immune-mediated component may be warranted, beyond the single study of celecoxib in ASD noted by Sethi et al. Reassuring safety data exist for both celecoxib and non-selective NSAIDs, derived from studies of children with juvenile idiopathic arthritis (51) and familial adenomatous polyposis (52). This approach may be particularly relevant in OCD given that the majority of affected individuals experience disease onset in childhood or adolescence, with a persistence rate of approximately 40% (53). Clinical practice guidelines suggesting the use of celecoxib as a third-line agent in adults with OCD (54) and naproxen or celecoxib in children with PANS/PANDAS (35) provide a further clinical imperative for these studies.

## Conclusion

Multiple lines of evidence suggest that aberrant inflammatory processes contribute to the pathogenesis of psychiatric disorders. Altered immune homeostasis may represent the consequence of exposure to environmental factors including psychosocial stress together with cumulative genetic and epigenetic risk. Changes in neuroendocrine regulation, metabolism, gut microbiota, and health behaviours in turn affect peripheral and central immune cell phenotypes. For individuals with the most severe symptoms refractory to traditional treatments, modulation of the innate immune system with COX-2 inhibitors appears to be an attractive—though understudied—therapeutic approach.

In characterizing state and trait markers of disease and identifying appropriate patients for anti-inflammatory treatments, broad immunophenotyping is likely to be essential. Moreover, preclinical studies suggesting effects of COX-2 inhibition on neurotransmitter function would suggest that traditional markers of inflammation in the periphery may not be required for therapeutic effect. The implications of differences in COX selectivity as well as COX-independent effects of individual NSAIDs in the CNS require further study. Finally, stressful events in childhood drive peripheral inflammation and affect neurodevelopment. Given our increasing understanding of innate immune memory and its potential role in neurodevelopment and neurodegeneration, the likely bidirectional relationship between inflammation and psychiatric symptoms, and the known benefits of early intervention, treatment trials of COX-2 inhibitors in carefully-selected pediatric populations are warranted.

## Author Contributions

CW-R conceived of and drafted the article. SES provided critical feedback and reviewed the final version to be submitted.

## Funding

Work by SES is supported by the Canadian Institutes of Health Research and Michael Smith Foundation for Health Research. CW-R is the recipient of a 2019 International OCD Foundation Young Investigator Award.

## Conflict of Interest

The authors declare that the research was conducted in the absence of any commercial or financial relationships that could be construed as a potential conflict of interest.
